# An Efficient Prediction Model on the Operation Quality of Medical Equipment Based on Improved Sparrow Search Algorithm-Temporal Convolutional Network-BiLSTM

**DOI:** 10.3390/s24175589

**Published:** 2024-08-29

**Authors:** Zicong Lin, Zhiyong Ji

**Affiliations:** College of Engineering Science and Technology, Shanghai Ocean University, Shanghai 201306, China

**Keywords:** MRI equipment, CT equipment, TCN-BiLSTM, ISSA, time series analysis

## Abstract

Combining medical IoT and artificial intelligence technology is an effective approach to achieve the intelligence of medical equipment. This integration can address issues such as low image quality caused by fluctuations in power quality and potential equipment damage, and this study proposes a predictive model, ISSA-TCN-BiLSTM, based on a bi-directional long short-term memory network (BiLSTM). Firstly, power quality data and other data from MRI and CT equipment within a 6-month period are collected using current fingerprint technology. The key factors affecting the active power of medical equipment are explored using the Pearson coefficient method. Subsequently, a Temporal Convolutional Network (TCN) is employed to conduct multi-layer convolution operations on the input temporal feature sequences, enabling the learning of global temporal feature information while minimizing the interference of redundant data. Additionally, bidirectional long short-term memory (BiLSTM) is integrated to model the intermediate active power features, facilitating accurate prediction of medical equipment power quality. Additionally, an improved Sparrow Search Algorithm (ISSA) is utilized for hyperparameter optimization of the TCN-BiLSTM model, enabling optimization of the active power of different medical equipment. Experimental results demonstrate that the ISSA-TCN-BiLSTM model outperforms other comparative models in terms of RMSE, MSE, and R2, with values of 0.1143, 0.1157, 0.0873, 0.0817, 0.95, and 0.96, respectively, for MRI and CT equipment. This model exhibits both prediction speed and accuracy in power prediction for medical equipment, providing valuable guidance for equipment maintenance and diagnostic efficiency enhancement.

## 1. Introduction

Medical equipment plays a vital role in supporting and ensuring the order and quality of diagnosis and treatment in hospitals [1]. In particular, large-scale medical equipment, such as computed tomography (CT) and magnetic resonance imaging (MRI), has greatly improved the level of medical care [2]. However, this medical equipment, embedded within complex operating systems and components sensitive to factors such as power quality, can cause distortions in medical images and even equipment failures because of fluctuations in active power, frequency interference, and electromagnetic interference, thereby affecting clinical diagnosis and treatment [3]. The occurrence of downtime in medical equipment can lead to unpredictable medical accidents and economic losses. Therefore, it is imperative for hospitals to ensure the reliability and operational excellence of their medical equipment. This is crucial to prevent interruptions, shutdowns, and other malfunctions that could compromise patient safety and the quality of diagnosis and treatment.

Currently, hospitals primarily enhance the reliability and efficiency of medical equipment through manual inspections and routine maintenance aligned with manufacturers’ guidelines. Nevertheless, as medical technology evolves and the quantity of medical equipment in contemporary hospitals surges, this approach is leading to inefficiencies, escalating costs, and delayed response times. Moreover, such traditional maintenance methods are often inadequate for predicting equipment abnormalities or sudden failures [4]. At the same time, the development of emerging technologies, such as the Internet of Things (IoT) and artificial intelligence (AI) is driving the innovation of medical equipment maintenance techniques [5,6,7]. IoT technology enables real-time monitoring of various electrical quality parameters (such as voltage, current, and active power) of medical equipment during operation and standby, as well as environmental characteristics (such as temperature and humidity) of the equipment’s working space by deploying sensors. The data from the equipment is then transmitted to a cloud platform through wireless technologies, such as Wi-Fi or cellular networks (4G/5G), and wired methods, such as ethernet or optical fiber. Finally, predictive models for medical equipment’s power quality are established based on artificial intelligence techniques such as machine learning and deep learning [8,9]. Machine learning-based predictive maintenance [10,11,12,13] strategies have already seen widespread implementation across various industries, including the mechanical and power sectors. These innovative approaches leverage data analysis and predictive algorithms to anticipate equipment failures before they occur, improving efficiency and reducing downtime. Dai et al. [14] introduced an intelligent diagnostic technique utilizing a multiscale gated convolutional neural network (MGCNN) specifically for diagnosing wear in sliding bearings. This approach offers robust support for monitoring the condition of equipment and enhances the capabilities of predictive maintenance. Lin et al. [15] enhanced the operational performance of power lines by employing advanced algorithms, such as support vector regression (SVR), gradient boosting regression (GBR), and long short-term memory neural networks (LSTM), which were instrumental in predicting leakage current in insulators with greater accuracy. However, predictive maintenance strategies for medical equipment are still in the early stages of development. Wang [3] proposed a multivariate time series classification model to predict abnormal conditions in CT equipment, using a sliding time window technique, which constructs new features over different time intervals. This approach helps reduce the interference of other factors on the predictive results. Spahić et al. [16] proposed a method based on artificial neural networks and fuzzy logic classifiers to predict the performance of infant incubators for upgrading medical equipment management strategies in healthcare institutions to address challenges of increased sophistication of equipment as well as patient safety demands.

The accurate prediction of active power of medical equipment is of great significance for identifying the operating status of the equipment. Due to the non-linear, dynamic, and uncertain characteristics of active power output in medical equipment, a combination of neural networks and optimization algorithms is necessary to enhance prediction accuracy [17]. To precisely identify the operating status of medical equipment and advance the development of predictive maintenance strategies for medical equipment, this paper introduces a medical equipment active power prediction model based on ISSA-TCN-BiLSTM. The input features play a crucial role in determining prediction accuracy [18]. The Pearson coefficient method is utilized to select key factors that influence changes in active power, enabling the model to effectively learn the underlying relationships within the data. Moreover, to overcome TCN-BiLSTM’s vulnerability to local optima when dealing with a large number of features in predicting active power for medical equipment, an enhanced Sparrow Search Algorithm is employed to search for optimal hyperparameters, thereby improving the reliability and stability of the predictive model.

## 2. Materials and Methods

### 2.1. Prediction Methods for Equipment Power

**Figure 1:**
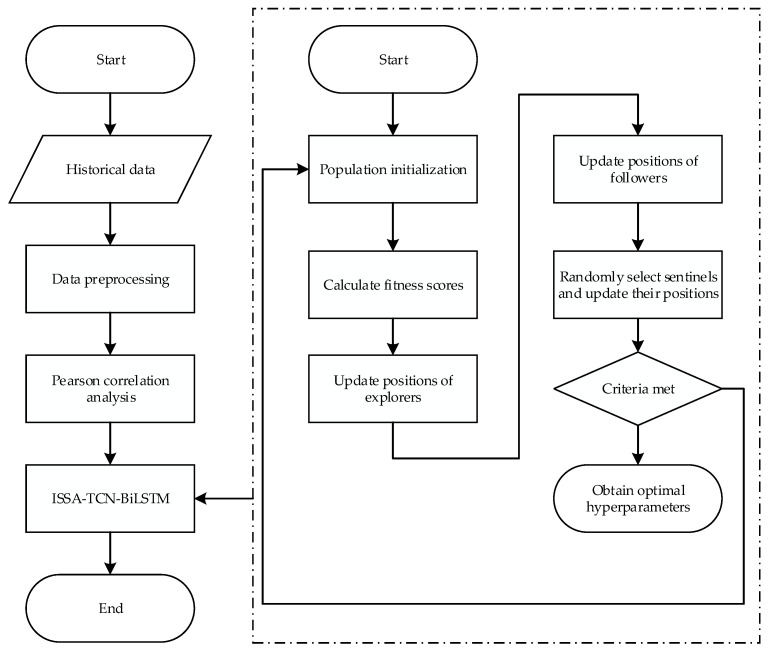
Prediction process of ISSA-TCN-BiLSTM.

An active power prediction model for medical equipment is developed utilizing the improved Sparrow Search Algorithm (ISSA) and the TCN-BiLSTM algorithm. This model comprises four key components: data preprocessing, Pearson correlation analysis, data prediction, and hyperparameter optimization. The complete prediction workflow is depicted in Figure 1.

Initially, intelligent equipment, such as dynamic energy identifiers and dynamic environment sensors, can gather power quality data from medical equipment and environmental information from within the equipment rooms. In order to improve the prediction accuracy of the model, this paper uses the K-nearest neighbor (KNN) method to complete the missing data and the pauta criterion to eliminate the abnormal data. KNN is a powerful technique that allows us to impute missing values based on the similarity of data points, maintaining the integrity of the dataset. Subsequently, the classification sample set is normalized to mitigate the impact of varying dimensions on the outcomes. Pearson correlation coefficients are used to pinpoint and choose features that significantly affect active power, thereby enhancing the quality of the training data for the active power prediction models. Finally, the refined training set is inputted into the TCN-BiLSTM model for training, with the ISSA being utilized to fine-tune the model’s hyperparameters.

### 2.2. TCN-BiLSTM

**Figure 2:**
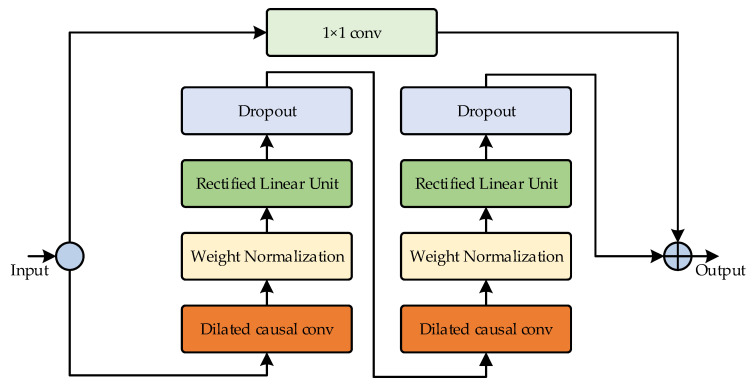
Residual block in TCN.

TCN is a time series data processing method based on convolutional neural networks [19]. Its main structure consists of a residual block containing dilated causal convolution. The residual block of TCN is illustrated in Figure 2.

TCN integrates residual blocks with dilated causal convolution, built on a foundation of one-dimensional convolution, to enhance the extraction of temporal features while also tackling the issue of vanishing or exploding gradients. Causal convolution is crucially utilized to ensure that predictions rely solely on past inputs, preventing future information leakage. Dilated convolution introduces interval sampling of inputs during convolution operations, effectively addressing the challenge of extracting information from multivariate time series data and expanding the receptive field. Additionally, employing the Rectified Linear Unit (ReLU) activation function and dropout techniques can significantly reduce overfitting, thereby improving the network’s learning speed and prediction accuracy.

The long short-term memory (LSTM) [20] neural network is a specialized variant of the recurrent neural network (RNN) [21], designed specifically to overcome the challenges of vanishing and exploding gradients that conventional RNNs encounter while processing sequential data. LSTM networks are widely used in time series scenarios that require long-term interval and time delay prediction.

LSTM network enhances the standard RNN framework by incorporating additional layers, which include memory cells and three distinct gates: the input gate, forget gate, and output gate. This structure enables selective information flow, with each gate performing a unique role in regulating the passage of data.

The forget gate is responsible for determining which information to discard from the storage unit. The calculation formula is as follows:

(1) $$f_{t}=\sigma \left(w_{f_{x}}x_{t}+w_{f_{h}}h_{t-1}+w_{f_{c}}C_{t-1}+b_{f}\right)$$

In the equation, $w_{f_{x}}$, $w_{f_{h}}$, $w_{f_{c}}$, and $b_{f}$ respectively denote the weight coefficients and bias for the forget gate.

The input gate is responsible for deciding which information to retain in the memory cell. The calculation formula is as follows:

(2) $$i_{t}=\sigma \left(w_{ix}x_{i}+w_{ih}h_{t-1}+w_{ic}C_{t-1}+b_{i}\right)$$

(3) $${\tilde{C}}_{t}=tanh\left(w_{cx}x_{t}+w_{ch}h_{t-1}+b_{O}\right)$$

(4) $$C_{t}=f_{t}\cdot C_{t-1}+i_{t}\cdot {\tilde{C}}_{t}$$

In the equation, $w_{ix}$, $w_{ih}$, $w_{ic}$, and $b_{i}$ respectively denote the weight coefficients and bias for the forget gate, and $w_{cx}$, $w_{ch}$, and $b_{O}$ respectively denote the weight coefficients and bias for the forget gate.

The output gate determines which information will be outputted. No other information can pass through the output gate besides what is required. The update equation is as follows:

(5) $$o_{t}=\sigma \left(w_{ox}x_{t}+w_{oh}h_{t-1}+w_{oc}C_{t-1}+b_{o}\right)$$

(6) $$h_{t}=o_{t}tanh\left(C_{t}\right)$$

In the equation, $i_{t}$, $f_{t}$ and $o_{t}$ respectively denote the input gate, forget gate and output gate, and $x_{t}$ represents the input at time t. The sigmoid activation function is represented by $\sigma \left(\cdot \right)$, and $tanh\left(\cdot \right)$ represents the hyperbolic tangent activation function. The weight coefficients of the output gate are denoted by $w_{ox}$, $w_{oh}$, $w_{oc}$, and $C_{t}$ and ${\tilde{C}}_{t}$ represent the candidate vector and the update value of the candidate vector at time t, respectively. The outputs at times t and (t − 1) are represented by $h_{t}$ and $h_{t-1}$, respectively.

**Figure 3:**
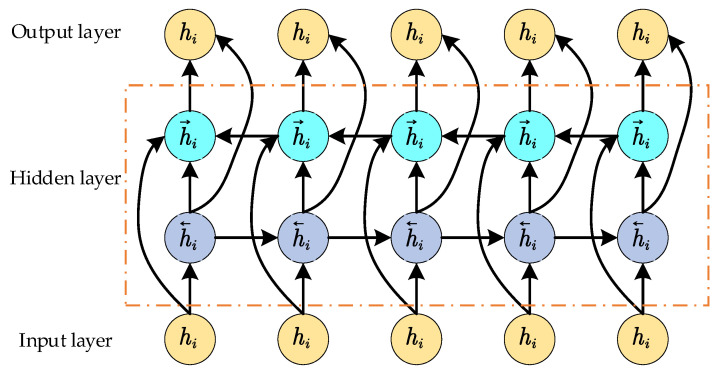
The structure of the BiLSTM network.

BiLSTM [22] enhances the network’s capacity to capture temporal sequence information by overlaying two independent LSTM layers, enabling the simultaneous acquisition of both past and future contextual information. The structure of BiLSTM is illustrated in Figure 3.

The forward and backward LSTM networks compute sequence information for forward and reverse input, respectively, yielding hidden state outputs of $\vec{H}$ and $\overset{\leftarrow }{H}$, which are then concatenated to produce the final output of the BiLSTM network layer. The specific calculation process is shown in Equations (7)–(9).

(7) $$\vec{H}=\left\{h_{L_{1}},h_{L_{2}},\cdots ,h_{L_{t}}\right\}$$

(8) $$\overset{\leftarrow }{H}=\left\{h_{R_{t}},h_{R_{t-1}},\cdots ,h_{R_{1}}\right\}$$

(9) $$H=\left\{\vec{H},\overset{\leftarrow }{H}\right\}$$

**Figure 4:**
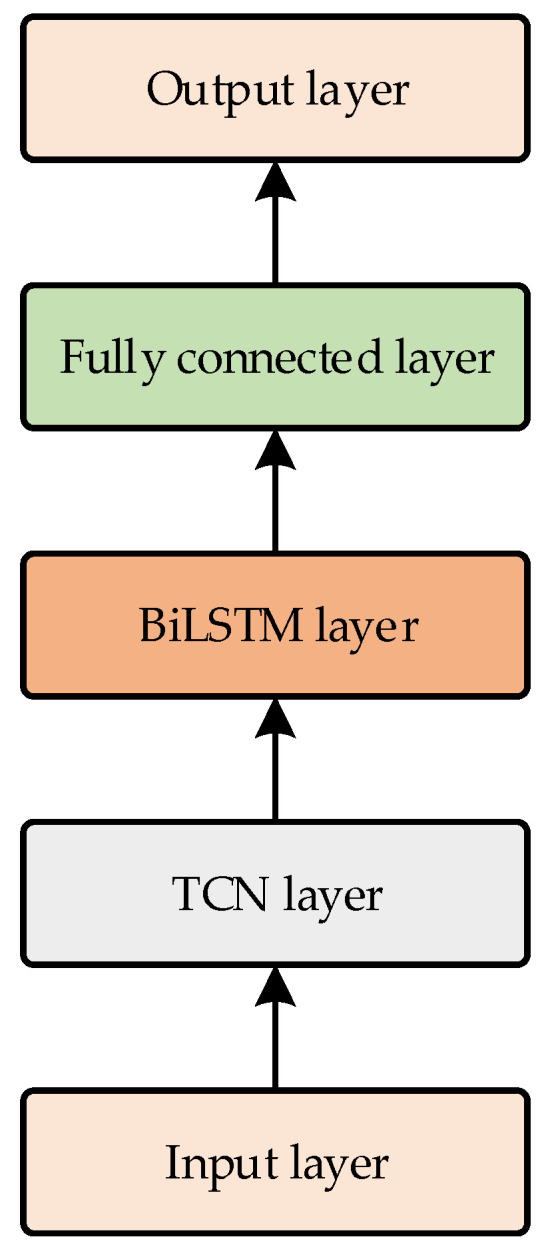
Architecture of TCN-BiLSTM.

This study combines the advantages of TCN and BiLSTM to construct a predictive model for the active power sequence of medical equipment, with its architecture depicted in Figure 4.

The model first uses a stack of three TCN residual blocks to capture a broader receptive field of the input sequence and to conduct feature extraction and dimensionality reduction. To prevent gradient explosion and vanishing, each residual block maintains a consistent kernel size, denoted as k. Unlike traditional convolution, dilated convolution samples input features at specified intervals, with the sampling rate determined by the dilation factor D. The effective sampling window of dilated convolution increases exponentially with the number of convolutional layers. This approach enables the model to achieve a large receptive field while utilizing fewer convolutional layers. Consequently, the dilation factors D are set to 1, 2, and 4, respectively. The BiLSTM layer, receiving the data sequence processed by the TCN, concatenates two LSTM layers that operate in opposite temporal directions—one forward and one backward. This approach enables BiLSTM to more comprehensively capture temporal dependencies and contextual associations. Finally, a fully connected layer maps the high-dimensional features to the final prediction outcome.

### 2.3. SSA

The Sparrow Search Algorithm (SSA) [23], introduced by Donghua University researchers in 2020, draws inspiration from the intricate behaviors of sparrows as they forage and evade predators. The SSA delineates the sparrow population into distinct roles: discoverers, followers, and vigilantes. Each group updates its position according to a specialized formula that reflects its function. When vigilantes perceive a threat, they signal an alarm, and if the danger level crosses a predefined threshold, the discoverers lead the followers to a safer location. This process operates iteratively, maintaining a stable ratio of discoverers to followers [24,25]. Simultaneously, followers, motivated by hunger, may embark on expeditions to uncharted territories in search of more energy-dense food. In addition, followers may enter a state of competition with the discoverers, who have secured the most nutritious food, thereby increasing their own foraging efficiency.

In the SSA, discoverers determine the search direction for the population, and their position updates during iterations are described by the Equations (10) and (11). If $R_{2}<s$, it implies a safe environment, facilitating a global search. Conversely, if $R_{2}⩾s$, it indicates the presence of danger, prompting the discoverers to adjust their positions.

(10) $$X_{i,j}^{t+1}=X_{i,j}^{t}\cdot exp\left(\frac{-i}{\alpha \cdot n_{max}}\right),R_{2}<s$$

(11) $$X_{i,j}^{t+1}=X_{i,j}^{t}+Q\cdot L,R_{2}⩾s$$

In Equations (10) and (11), $X_{i,j}$ denotes the positional information of the sparrows, t denotes the number of iterations, $\alpha \in \left(0,1\right]$ is a random number, $n_{max}$ represents the maximum number of iterations, $R_{2}\in \left[0,1\right]$ denotes the threshold value, $s\in \left[0.5,1\right]$ signifies the safety value, Q is a random variable following a standard normal distribution, and L represents a 1 × d matrix.

The follower’s position update is described as per the Equations (12) and (13), indicating that for i>n/2, the ith follower is in a poorer state, necessitating the search for a new position.

(12) $$X_{i,j}^{t+1}=Q\cdot exp\left(\frac{X_{worst}^{t}-X_{i,j}^{t}}{i^{2}}\right),i>n/2$$

(13) $$X_{i,j}^{t+1}=X_{P}^{t+1}+\left|X_{i,j}^{t}-X_{P}^{t+1}\right|\cdot A^{+}\cdot L,i⩽n/2$$

In Equations (12) and (13), $X_{worst}$ represents the current global worst position, $X_{P}$ denotes the position of the best explorer, and A is a 1 × d matrix with each element randomly assigned a value of 1 or −1.

When certain individuals sense danger, their positions undergo a jump, as depicted by the given Equations (14) and (15). If $f_{i}>f_{g}$, it indicates the individual is at the edge of the population; if $f_{i}=f_{g}$, it signifies that individuals in the middle of the population should move closer to the group to reduce risk.

(14) $$X_{i,j}^{t+1}=X_{best}^{t}+\beta \cdot \left|X_{i,j}^{t}-X_{best}^{t}\right|,f_{i}>f_{g}$$

(15) $$X_{i,j}^{t+1}=X_{i,j}^{t}+K\cdot \left[\frac{\left|X_{i,j}^{t}-X_{worst}^{t}\right|}{\left(f_{i}-f_{w}\right)+\varepsilon }\right],f_{i}=f_{g}$$

In Equations (14) and (15), $X_{best}^{t}$ denotes the current global optimum position, β signifies a normally distributed random number with mean 0 and variance 1, $f_{i}$ stands for fitness value, $f_{g}$ and $f_{w}$ represent the current best and worst fitness values, respectively, K is a random number, and ε is a nonzero constant.

### 2.4. Improved SSA

SSA, as a metaheuristic optimization algorithm, can be used to optimize the parameter selection of TCN-BiLSTM, reducing computation time and improving prediction accuracy. The Sparrow Search Algorithm is notably effective in tackling intricate optimization challenges. However, its reliance on a randomly generated initial population can result in an uneven distribution, which may restrict the diversity and quality of the population. This can impede the convergence rate of the algorithm [26]. Moreover, SSA’s performance may be further compromised by a lack of robust parameter control mechanisms, making it prone to getting trapped in local optima and experiencing reduced accuracy in subsequent iterations.

Opposition-based learning (OBL) [27] is a strategy that has gained significant recognition for its ability to enhance the diversity of populations within intelligent algorithms. By introducing the concept of opposing points and replacing random searches with adversarial searches, OBL effectively accelerates the convergence rate and strengthens the precision of solutions derived from algorithms. In this study, the fundamental concept behind utilizing the OBL strategy for population generation is to start by creating an initial random population. Subsequently, a corresponding opposing population is formed based on the initial one. The superior set from these two is then selected to be the progenitor of the next generation. The OBL strategy prioritizes individuals that are closer to the optimal solution than the initial members of the population. This strategic approach ensures that each individual begins closer to the optimal solution, thereby speeding up the convergence rate for the entire population. Moreover, OBL contributes to enhancing population diversity and fortifying the algorithm’s capability to conduct a more thorough global search by exploring more promising regions.

According to previous research [28], the step size control parameters β and K are two of the most important adjustable parameters in the SSA. Appropriate step size control parameters can balance the local and global search capabilities of the algorithm, thereby reducing the number of iterations required to locate the optimal solution and improving the performance of the SSA. However, due to the fact that the step size control parameters in the SSA are all random numbers, it is not possible for the discoverer to fully explore the space, which may lead to the algorithm falling into local optima. Due to the negative correlation between step size control parameters and the algorithm population diversity, in the early stages of algorithm iteration, the population has high diversity, which means that the ability to explore global space is strong and the ability to explore local space is weak. Therefore, in this study, the value of the step control parameter is set to a smaller value at first, in order to enhance the local exploration ability. In the later stage of iteration, when all sparrows are attracted to the global optimum and there is not enough search space, the algorithm tends to converge. At this time, the step size control parameter should be set to a larger value to avoid getting stuck in local optima. Therefore, dynamically adjusting the step size control parameters in this study can balance the global and local search capabilities of the SSA, while improving model prediction accuracy and avoiding the occurrence of local optimal solutions. The improved step size control parameters β and K are shown in Equations (16) and (17):

(16) $$\beta =f_{g}-\left(f_{g}-f_{w}\right)\cdot {\left(\frac{n_{max}-t}{n_{max}}\right)}^{1.5}$$

(17) $$K=\left(f_{g}-f_{w}\right)\cdot exp\left(-20\cdot tan\left(\frac{t}{n_{max}}\right)\right)\cdot \left(2\cdot r-1\right)$$

In the equation, $f_{g}$ and $f_{w}$ represent the current best and worst fitness values, $n_{max}$ represents the maximum number of iterations, and $r\in \left[0,1\right]$ is a random number.

In order to further reduce the possibility of the SSA falling into local optima, which would result in an inability to find the global optimal solution, the Levy flight strategy [29] is introduced to enhance the local search ability of the SSA. This enhancement effectively solves the problem of the SSA falling into local optima. The improved SSA updates the position based on the distance between the current position and the sparrow’s optimal position, significantly reducing the risk of sparrows falling into local optima. Equation (14) is updated to Equation (18).

(18) $$X_{i,j}^{t+1}=Levy\left(d\right)\cdot X_{best}^{t}+\beta \cdot \left|X_{i,j}^{t}-Levy\left(d\right)\cdot X_{best}^{t}\right|,f_{i}>f_{g}$$

In the equation, d represents the spatial dimension. The Lévy calculation formula is shown in Equations (19) and (20).

(19) $$Levy\left(d\right)=0.01\cdot \frac{\gamma_{1}\cdot \sigma }{{\left|\gamma_{2}\right|}^{1/\beta }}$$

(20) $$\sigma ={\left\{\frac{\Gamma \left(1+\theta \right)\cdot sin\left(\pi \theta /2\right)}{\Gamma \left[\left(1+\lambda \right)/2\right]\cdot \theta \cdot 2^{\left(\theta -1\right)/2}}\right\}}^{1/\theta }$$

In the equation, Γ represents the Gamma function, θ is a constant, and $\gamma_{1}$ and $\gamma_{2}$ are random numbers in the range [0, 1].

## 3. Experimental and Results Analysis

### 3.1. Explanation of Experimental Data

The experimental data used in this study were obtained from the active power readings, frequency of use, operational status, spatial environmental parameters of the equipment room (temperature, humidity, and cleanliness), and the maintenance frequency of MRI and CT equipment at a hospital in East China from June to December 2023. The data consist of active power measurements, equipment parameters, and spatial environmental parameters of the equipment room gathered from the equipment rooms with MRI and CT equipment. The data was sampled every 10 min within the time windows of 08:00–16:00; 48 data were obtained every day, and a total of 10,224 data records were collected for active power prediction on 5 and 6 January 2024. In the sample of 213 d, the ratio of training sets and test sets is 7:3. The seven influence factors and active power values of historical data are taken as input vectors of the prediction model, and the output result of the model is the active power value of the forecast day. After conducting a Pearson correlation analysis between the collected data and power, data with high correlation (including equipment parameters, operational status, temperature, humidity, equipment cleanliness, and maintenance frequency) were retained. These variables also served as input parameters for the network. The data characteristics of these influencing factors are shown in Table 1.

### 3.2. Data Preprocessing

#### 3.2.1. Anomaly Data Processing

During the collection of historical meteorological data and active power data, storage device faults and network signal transmission fluctuations may affect the sample data, resulting in outliers and missing values. In order to improve the effectiveness and availability of the collected data and realize the accurate prediction of the active power of medical equipment, the data collected in this period is preprocessed every 2 h. To enhance the model’s predictive accuracy, this study utilizes the KNN method to impute missing data and applies the pauta criterion to eliminate outlier data. As shown in the Equation (21), KNN first calculates the Euclidian distance $d_{i}(X_{i},X_{j})$ between the target active power data (data records with missing items) and all fully valued data records in the data set.

(21) $$d_{i}(X_{i},X_{j})=\sqrt{\sum_{r=1}^{m}{\left(x_{ir}-x_{jr}\right)}^{2}}$$

In the equation, $X_{i}=\left\{x_{i1},x_{i2},\cdots ,x_{im}\right\}$ represents the first m-dimensional data of the ith sample, and $x_{ir}$ denotes the rth dimensional attribute of the ith sample.

Secondly, the k data records with the smallest Euclidian distance from the target data are selected as the nearest neighbors of the target data. As shown in the formula, the weight of the nearest neighbor of the target data is as follows:

(22) $$w_{i}=\frac{1/d_{i}}{\sum_{i=1}^{k}1/d_{i}}$$

Finally, the weighted average value of the corresponding position value recorded by k’s nearest neighbor data is the estimated value of the missing active power data:

(23) $$\tilde{g}=\sum_{i=1}^{k}w_{i}x_{i}$$

In the equation, $w_{i}$ represents the value of the nearest neighbor corresponding position.

The equation for pauta criterion is as follows:

(24) $$∆x_{i}>3\sqrt{\sum_{i=1}^{n}\frac{∆x_{i}^{2}}{n-1}}$$

In the equation, $∆x_{i}=x_{i}-{\bar{x}}_{i}$. If the condition is met, $x_{i}$ is identified as an outlier and will be replaced using the K-nearest neighbors method.

#### 3.2.2. Data Normalization

To expedite model training, the raw sequential data is normalized to compress the value range of all features between zero and one, maintaining the variance and distribution while preventing excessively large values from disproportionately impacting the model. The equation of data normalization is as follows:

(25) $$x^{\prime }=\frac{x-x_{min}}{x_{max}-x_{min}}$$

In the equation, $x^{\prime }\in [0,1]$ denotes the normalized data, and $x_{min}$ and $x_{max}$ represent the minimum and maximum values in the dataset, respectively.

### 3.3. Model Evaluation Metrics

To assess the predictive performance of the model, the study used the following metrics: mean squared error (MSE), mean absolute error (MAE), R-squared (R^2^), and run time (RT). Lower values of MSE and MAE, along with a higher R^2^, signify enhanced predictive accuracy. While upholding predictive accuracy, a reduced RT signifies superior model performance. The equations for the respective evaluation metrics are as follows:

(26) $$e_{RMSE}=\sqrt{\frac{1}{n}\sum_{i=1}^{n}{\left(P_{i}-{\overset{̑}{P}}_{i}\right)}^{2}}$$

(27) $$e_{MAE}=\frac{1}{n}\sum_{i=1}^{n}\left|P_{i}-{\overset{̑}{P}}_{i}\right|$$

(28) $$e_{RS}=\left[1-\frac{\sum_{i=1}^{n}{\left(P_{i}-{\overset{̑}{P}}_{i}\right)}^{2}}{\sum_{i=1}^{n}{\left(P_{i}-\bar{P}\right)}^{2}}\right]$$

In the equations, n denotes the dimension of the power series in the test set, $P_{i}$ represents the actual power, ${\overset{̑}{P}}_{i}$ is the predicted power, and $\bar{P}$ is the mean of the actual power values.

### 3.4. Experiment Platform and Training Hyperparameters

In this study, the improved model is used on a deep learning server with the configuration shown in Table 2.

Some of the training hyperparameters of the ISSA-TCN-BiLSTM model are as follows: The convolution kernel size is 3; the expansion coefficient D is 1,2,4; the dropout probability in TCN is 0.1; the number of BiLSTM layers is 2; and the total iteration number is 300. The selection of ISSA parameters is based on a large number of experiments and references, and these values provide the best evaluation indicators and the best computational efficiency on the training data set. The training hyperparameters of the improved SSA are shown in Table 3.

### 3.5. Model Comparison Analysis

**Figure 5:**
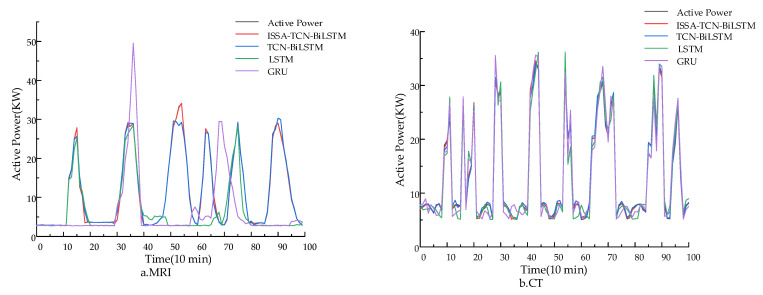
Comparison of prediction performance of different models.

**Figure 6:**
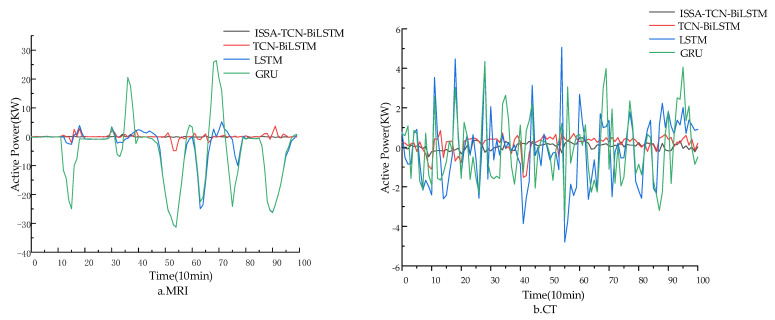
Comparisons of prediction errors among different models.

To verify the predictive performance of the proposed model, this study compares it with various other forecasting models. ISSA-TCN-BiLSTM is compared against models such as the Gated Recurrent Unit (GRU) [30], LSTM networks, and TCN-BiLSTM forecasting models. In order to verify the predictive performance of the model for active power of different medical equipment, 5 January 2024, was selected as the prediction day to predict the active power of MRI and CT equipment, as shown in Figure 5. Figure 5 displays the prediction curves of active power for MRI and CT equipment using four different models. As the medical equipment transitions from standby to startup, there is a slight increase and fluctuation in active power. When the medical equipment enters operating mode, the active power suddenly increases. Meanwhile, Figure 6 illustrates the prediction errors corresponding to different models. The gating mechanism in LSTM and the GRU allows the model to selectively receive or ignore input information; thus, it still has a good prediction effect during the time period when the operating state of the equipment changes, and then the change of active power data shows an inflection point. The GRU and LSTM cannot effectively extract the variation characteristics of the active power of the equipment, as the operating state of the equipment changes frequently. It can be seen from Figure 6 that the ISSA-TCN-BiLSTM proposed in this paper has the smallest error curve fluctuation compared to other models. This is because the active power of medical equipment is influenced by various key factors, such as operating conditions, computer room environment, usage frequency, and maintenance frequency. Preprocessing the dataset before prediction can optimize the input samples. The TCN-BiLSTM combination model can merge the advantages of different algorithms to effectively learn long-term dependencies between data while extracting feature information with a larger receptive field range, reducing the risk of overfitting. In addition, using the ISSA to optimize the parameter selection required for the TCN-BiLSTM model can improve the search speed of the model, overcome the blindness and limitations of the traditional LSTM model in parameter selection, and thus improve prediction accuracy.

To analyze the differences among the models, a comparative analysis of RMSE, MAE, R2, and RT for the four models was undertaken (Model 1 represents LSTM; Model 2 represents GRU; Model 3 represents TCN-BiLSTM; Model 4 represents ISSA-TCN-BiLSTM), the results are shown in Table 4. The ISSA-TCN-BiLSTM model achieved an RMSE of 0.1143 and an MAE of 0.1157 on MRI equipment, and an RMSE of 0.1157 and MAE of 0.0817 on CT equipment. The improved model attained R2 values of 0.95 and 0.96 on different equipment. The proposed method significantly outperforms mainstream forecasting models in evaluation metrics, especially with a runtime of only 3.9864 s and 3.8712 s, indicating high real-time performance.

The experimental results demonstrate that the model proposed in this article exhibits robust learning capabilities when faced with abrupt changes in operating state samples. Additionally, it achieves superior generalization performance, thereby minimizing operational errors during equipment operating state transitions. Notably, ISSA-TCN-BiLSTM demonstrates excellent predictive performance across various medical equipment types and serves as an effective method for forecasting the active power of medical equipment. Consequently, it can promptly and precisely predict the operating status of medical equipment.

## 4. Discussion

In this study, a predictive model for active power of medical equipment is proposed based on an improved Sparrow Search Algorithm (ISSA), Temporal Convolutional Network (TCN), and Bi-directional long short-term memory network (BiLSTM). The experimental results demonstrate that this model outperforms GRU, LSTM, and other existing methods in terms of accuracy and error in active power prediction. The model addresses issues related to missing data and poor robustness when the operating state of medical equipment changes, and it can meet the prediction requirements for active power of various medical equipment.

The ISSA-TCN-BiLSTM model extracts and reduces the dimension of active power features by combining TCN and BiLSTM to prevent gradient explosion in active power prediction for medical equipment, which can hinder model convergence. By adjusting the dilation factor D, TCN can achieve a larger input time series receptive field with fewer convolutional layers, reducing calculation time and enhancing prediction accuracy. The BiLSTM model links positive and negative LSTM layers to better capture context information from time series data and improve the model’s ability to identify active power features. Additionally, ISSA is utilized to optimize the hyperparameters of the TCN-BiLSTM model, aiming to enhance the hyperparameters for active power prediction tasks across various medical equipment.

The experimental results demonstrate that the model proposed in this article exhibits robust learning capabilities when faced with abrupt changes in operating state samples. Additionally, it achieves superior generalization performance, thereby minimizing operational errors during equipment operating state transitions. Notably, ISSA-TCN-BiLSTM demonstrates excellent predictive performance across various medical equipment types and serves as an effective method for forecasting the active power of medical equipment. Consequently, it can promptly and precisely predict the operating status of medical equipment. The ISSA-TCN-BiLSTM model strikes a good balance between the stability and accuracy of prediction, making it an effective method for predicting the active power of medical equipment.

Although ISSA-TCN-BiLSTM shows strong performance, it also has certain limitations. The diversity and size of the dataset may affect the model’s generalization ability. Due to the lack of current medical equipment active power datasets, the model proposed in this paper has not been validated on external datasets. Therefore, in future work, we will incorporate transfer learning and multimodal technology in the application of other types of medical equipment for experimentation, exploration, and advancement. Simultaneously, we will conduct more comparisons between different models to facilitate a more in-depth analysis and discussion of the model’s generalization performance.

## 5. Summary

In order to accurately monitor the operational status of medical equipment, this paper proposes a medical equipment active power prediction model based on ISSA-TCN-BiLSTM. The main conclusions are as follows:

(1) Through Pearson correlation analysis, abnormal data processing, and normalization, the original sample set is preprocessed to extract useful features related to active power changes. This process enhances the correlation between input features, addressing the limitations of low input quality and high information redundancy in traditional prediction models.
(2) The use of TCN to extract and reduce key input features has improved the accuracy of BiLSTM. In addition, by improving the global search and local search capabilities of the SSA, the parameter selection of TCN-BiLSTM is optimized, aiming to avoid blind parameter settings and getting stuck in local optimal solutions. Verified by actual measurement data, the model proposed in this article can effectively improve prediction accuracy, thereby achieving reliable prediction of active power for different medical equipment.
(3) When using different evaluation criteria to measure model performance, some potential influencing factors are sometimes ignored. Therefore, in the final evaluation step of the model prediction performance, we selected the three evaluation criteria of RMSE, MAE, and R2 to evaluate the accuracy of the model. At the same time, RT was used to evaluate the complexity of the model by the speed of the model response. Through comparative experiments, when the ISSA-TCN-BiLSTM model proposed in this paper is applied to MRI and CT equipment, its RMSE, MAE, and R2 values show that its prediction performance is better than LSTM, GRU, and TCN-BiLSTM models, while the RT index is slightly inferior to LSTM and GRU, but in a large data set, this impact is small. Combining these four evaluation criteria helps to obtain a more objective and effective evaluation of the model proposed in this paper.

The ISSA-TCN-BiLSTM model proposed in this paper improves the accuracy of time series prediction of medical equipment monitoring points and provides a theoretical basis for decision-making on the preventive maintenance cycle of medical equipment and reducing maintenance costs. In future work, we will conduct an in-depth study of large-scale medical equipment active power prediction tasks. In addition, we will combine machine learning, deep learning, multi-modality, and transfer learning and build different medical equipment active power datasets to solve various medical equipment power prediction tasks.

## Figures and Tables

**Table 1:** Data features of input vector.

Input Vector	Data Features
Active power	The active power value of the medical equipment at the sampling time point, expressed in KW.
Frequency of use	Record of the frequency of the use of medical equipment, expressed as number of times.
Operational status	Record of the current operating state of the equipment. MRI equipment and CT equipment are divided into standby state and running state.
Temperature	The temperature change in the equipment room affects the stability of medical devices.
Humidity	The humidity change in the equipment room affects the stability of medical devices.
Equipment cleanliness	The cleanliness index of medical equipment is evaluated by scoring.
Maintenance frequency	Record the maintenance times of the medical equipment. Maintenance of medical equipment can improve the operation stability.
Equipment parameters	Includes models of medical equipment (MRI and CT) and power levels.

**Table 2:** Deep learning server configuration.

Configuration	Parameter
CPU	Inter(R) Xeon(R) W-2223 (Inter, Santa Clara, CA, USA)
GPU	Nvidia GeForce RTX 2080ti (Nvidia, Santa Clara, CA, USA)
Operating system	Windows 10
Interpreter setting	Python 3.8 and torch 1.13.1

**Table 3:** Training parameter.

Parameter	Value
Population size	50
Discoverer	20%
Followers	80%
Initial alerter	10%
Iterations	300

**Table 4:** Analysis of model forecast on 3 January 2024.

Model	RMSE		MAE		R^2^		RT	
	MRI	CT	MRI	CT	MRI	CT	MRI	CT
1	0.2435	0.2978	0.2246	0.2811	0.74	0.71	3.5456	3.9512
2	0.2105	0.2231	0.1873	0.1689	0.83	0.85	3.8954	4.1023
3	0.1925	0.1974	0.1561	0.1421	0.91	0.90	4.5489	4.6127
4	0.1143	0.1157	0.0873	0.0817	0.95	0.96	3.9864	4.1712

## Data Availability

Data are contained within the article.

## References

[1] Shamayleh A., Awad M., Farhat J. (2020). IoT Based Predictive Maintenance Management of Medical Equipment. J. Med. Syst..

[2] Abhisheka B., Biswas S.K., Purkayastha B., Das D., Escargueil A. (2024). Recent Trend in Medical Imaging Modalities and Their Applications in Disease Diagnosis: A Review. Multimed. Tools Appl..

[3] Wang C., Liu Q., Zhou H., Wu T., Liu H., Huang J., Zhuo Y., Li Z., Li K. (2023). Anomaly Prediction of CT Equipment Based on IoMT Data. BMC Med. Inform. Decis. Mak..

[4] Khalaf A.B., Hamam Y., Alayli Y., Djouani K. (2013). The Effect of Maintenance on the Survival of Medical Equipment. J. Eng. Des. Technol..

[5] Atzori L., Iera A., Morabito G. (2010). The Internet of Things: A Survey. Comput. Netw..

[6] Oniani S., Marques G., Barnovi S., Pires I.M., Bhoi A.K. (2021). Artificial Intelligence for Internet of Things and Enhanced Medical Systems. Bio-Inspired Neurocomputing.

[7] Sun L., Jiang X., Ren H., Guo Y. (2020). Edge-Cloud Computing and Artificial Intelligence in Internet of Medical Things: Architecture, Technology and Application. IEEE Access.

[8] Al-Turjman F., Nawaz M.H., Ulusar U.D. (2020). Intelligence in the Internet of Medical Things Era: A Systematic Review of Current and Future Trends. Comput. Commun..

[9] Mohanta B., Das P., Patnaik S. Healthcare 5.0: A Paradigm Shift in Digital Healthcare System Using Artificial Intelligence, IOT and 5G Communication. Proceedings of the 2019 International Conference on Applied Machine Learning (ICAML).

[10] Bezerra F.E., Oliveira Neto G.C.d., Cervi G.M., Francesconi Mazetto R., Faria A.M.d., Vido M., Lima G.A., Araújo S.A.d., Sampaio M., Amorim M. (2024). Impacts of Feature Selection on Predicting Machine Failures by Machine Learning Algorithms. Appl. Sci..

[11] Arafat M.Y., Hossain M.J., Alam M.M. (2024). Machine Learning Scopes on Microgrid Predictive Maintenance: Potential Frameworks, Challenges, and Prospects. Renew. Sustain. Energy Rev..

[12] Li Z., He Q., Li J. (2024). A Survey of Deep Learning-Driven Architecture for Predictive Maintenance. Eng. Appl. Artif. Intell..

[13] Dehghan Shoorkand H., Nourelfath M., Hajji A. (2024). A Hybrid CNN-LSTM Model for Joint Optimization of Production and Imperfect Predictive Maintenance Planning. Reliab. Eng. Syst. Saf..

[14] Dai J., Tian L., Chang H. (2024). An Intelligent Diagnostic Method for Wear Depth of Sliding Bearings Based on MGCNN. Machines.

[15] Lin Y.-T., Kuo C.-C. (2024). Real-Time Salt Contamination Monitoring System and Method for Transmission Line Insulator Based on Artificial Intelligence. Appl. Sci..

[16] Spahić L., Kurta E., Ćordić S., Bećirović M., Gurbeta L., Kovacevic Z., Izetbegovic S., Badnjevic A. (2020). Machine Learning Techniques for Performance Prediction of Medical Devices: Infant Incubators. Proceedings of the CMBEBIH 2019.

[17] Akkaya S., Yüksek E., Akgün H.M. (2024). A Comprehensive Research of Machine Learning Algorithms for Power Quality Disturbances Classifier Based on Time-Series Window. Electr. Eng..

[18] Vouk B., Guid M., Robnik-Šikonja M. (2023). Feature Construction Using Explanations of Individual Predictions. Eng. Appl. Artif. Intell..

[19] Bai S., Kolter J.Z., Koltun V. (2018). An Empirical Evaluation of Generic Convolutional and Recurrent Networks for Sequence Modeling. arXiv.

[20] Graves A. (2012). Long Short-Term Memory. Supervised Sequence Labelling with Recurrent Neural Networks.

[21] Shahid F., Zameer A., Muneeb M. (2021). A Novel Genetic LSTM Model for Wind Power Forecast. Energy.

[22] Siami-Namini S., Tavakoli N., Namin A.S. The Performance of LSTM and BiLSTM in Forecasting Time Series. Proceedings of the 2019 IEEE International Conference on Big Data (Big Data).

[23] Xue J., Shen B. (2020). A Novel Swarm Intelligence Optimization Approach: Sparrow Search Algorithm. Syst. Sci. Control Eng..

[24] Wu Z., Wang B. (2021). An ensemble neural network based on variational mode decomposition and an improved sparrow search algorithm for wind and solar power forecasting. IEEE Access.

[25] Wang Z., Ying Y., Kou L., Ke W., Wan J., Yu Z., Liu H., Zhang F. (2024). Ultra-Short-Term Offshore Wind Power Prediction Based on PCA-SSA-VMD and BiLSTM. Sensors.

[26] Blazakis K., Schetakis N., Bonfini P., Stavrakakis K., Karapidakis E., Katsigiannis Y. (2024). Towards Automated Model Selection for Wind Speed and Solar Irradiance Forecasting. Sensors.

[27] Tizhoosh H.R. Opposition-Based Learning: A New Scheme for Machine Intelligence. Proceedings of the International Conference on Computational Intelligence for Modelling, Control and Automation and International Conference on Intelligent Agents, Web Technologies and Internet Commerce (CIMCA-IAWTIC’06).

[28] Hu L., Wang D. (2024). Research and Application of an Improved Sparrow Search Algorithm. Appl. Sci..

[29] Reynolds A. (2015). Liberating Lévy Walk Research from the Shackles of Optimal Foraging. Phys. Life Rev..

[30] Dey R., Salem F.M. Gate-Variants of Gated Recurrent Unit. (GRU) Neural Networks. Proceedings of the 2017 IEEE 60th International Midwest Symposium on Circuits and Systems (MWSCAS).

